# Exploring Differentially Methylated Genes in Vulvar Squamous Cell Carcinoma

**DOI:** 10.3390/cancers13143580

**Published:** 2021-07-16

**Authors:** Shatavisha Dasgupta, Patricia C. Ewing-Graham, Sigrid M. A. Swagemakers, Thierry P. P. van den Bosch, Peggy N. Atmodimedjo, Michael M. P. J. Verbiest, Marit de Haan, Helena C. van Doorn, Peter J. van der Spek, Senada Koljenović, Folkert J. van Kemenade

**Affiliations:** 1 Department of Pathology, Erasmus MC, University Medical Centre Rotterdam, 3000 CA Rotterdam, The Netherlands; p.ewing@erasmusmc.nl (P.C.E.-G.); s.swagemakers@erasmusmc.nl (S.M.A.S.); t.vandenbosch@erasmusmc.nl (T.P.P.v.d.B.); p.atmodimedjo@erasmusmc.nl (P.N.A.); m.haan.1@erasmusmc.nl (M.d.H.); p.vanderspek@erasmusmc.nl (P.J.v.d.S.); s.koljenovic@erasmusmc.nl (S.K.); f.vankemenade@erasmusmc.nl (F.J.v.K.); 2 Department of Clinical Bioinformatics, Erasmus MC, University Medical Centre Rotterdam, 3000 CA Rotterdam, The Netherlands; 3 Department of Internal Medicine, Erasmus MC, University Medical Centre Rotterdam, 3000 CA Rotterdam, The Netherlands; m.m.p.j.verbiest@erasmusmc.nl; 4 Department of Gynecologic Oncology, Erasmus MC Cancer Institute, University Medical Centre Rotterdam, 3000 CA Rotterdam, The Netherlands; h.vandoorn@erasmusmc.nl

**Keywords:** vulva, vulvar neoplasms, epigenomics, DNA methylation, differentially methylated genes, DNA copy number variations

## Abstract

**Simple Summary:**

Vulvar squamous cell carcinoma (VSCC) is the most common form of vulvar malignancy, and its incidence has increased in recent years. For better diagnosis and prognostication, and to expand available treatment options, molecular characterization of VSCC is crucial. We sought to identify aberrations in DNA methylation in VSCC, as this has been implicated in the development of several cancers. To this end, we performed genome-wide methylation sequencing on a set of VSCC and normal vulvar tissue using the Infinium MethylationEPIC BeadChip array. We detected 199 genes to be differentially methylated in VSCC compared to normal vulvar tissue. Of these, 194 genes were hyper-methylated, which leads to a loss of function of the genes. As most of these genes are involved in transcription regulator activity, our results suggest that disruption of this process plays an important role in VSCC development.

**Abstract:**

DNA methylation is the most widely studied mechanism of epigenetic modification, which can influence gene expression without alterations in DNA sequences. Aberrations in DNA methylation are known to play a role in carcinogenesis, and methylation profiling has enabled the identification of biomarkers of potential clinical interest for several cancers. For vulvar squamous cell carcinoma (VSCC), however, methylation profiling remains an under-studied area. We sought to identify differentially methylated genes (DMGs) in VSCC, by performing Infinium MethylationEPIC BeadChip (Illumina) array sequencing, on a set of primary VSCC (*n* = 18), and normal vulvar tissue from women with no history of vulvar (pre)malignancies (*n* = 6). Using a false-discovery rate of 0.05, beta-difference (Δβ) of ±0.5, and CpG-island probes as cut-offs, 199 DMGs (195 hyper-methylated, 4 hypo-methylated) were identified for VSCC. Most of the hyper-methylated genes were found to be involved in transcription regulator activity, indicating that disruption of this process plays a vital role in VSCC development. The majority of VSCCs harbored amplifications of chromosomes 3, 8, and 9. We identified a set of DMGs in this exploratory, hypothesis-generating study, which we hope will facilitate epigenetic profiling of VSCCs. Prognostic relevance of these DMGs deserves further exploration in larger cohorts of VSCC and its precursor lesions.

## 1. Introduction

Vulvar squamous cell carcinoma (VSCC) is considered to be a rare cancer; however, an increase in the incidence and a decrease in the median age of onset has been witnessed over recent decades [1,2,3,4]. VSCC comprises two main etiological subtypes–human papillomavirus (HPV)-associated and HPV-independent [5,6]. These subtypes are also known to differ in their epidemiological, clinical, pathological, and molecular characteristics. For example, HPV-associated VSCC, the less prevalent subtype, affects women of 50–60 years of age, and is associated with a favorable prognosis [7,8]. In contrast, the more prevalent HPV-independent VSCC usually develops on the background of chronic dermatoses in women of >70 years of age, and is associated with high rates of recurrence [2,5,6,7,9]. On a molecular level, somatic mutations of *TP53* have been frequently detected in HPV-independent VSCCs, and have been implicated as the ‘oncogenic driver’ for this subtype [7,10,11,12]. However, recent studies have discovered molecular heterogeneity among HPV-independent VSCCs, as some of these tumors can be *TP53* wild-type, and harbor *HRAS* or *NOTCH1* mutations instead [7,10].

Although our understanding of VSCC carcinogenesis has progressed, treatment options for VSCC patients have not significantly evolved over the years [13,14]. The mainstay of VSCC treatment remains surgery with tumor-free resection margins, confirmed on microscopy [13,14,15]. Unfortunately, surgical interventions in the vulva may injure adjacent vital structures such as the urethra or the anus, resulting in post-operative morbidity and a reduced quality of life [16]. Effective therapeutic alternatives that may help avoid such adverse consequences are therefore an urgent unmet need.

For the identification of biomarkers of potential diagnostic, prognostic, predictive, or therapeutic interest, comprehensive molecular characterization of cancer, by comparing molecular profiles of cancer and normal tissue from the same anatomical site is essential [17]. In recent years, several studies have investigated genomic changes, e.g., somatic mutations and copy number variations in VSCC, and its precursor lesion, vulvar intraepithelial neoplasia (VIN) [7,10,11,12,18]. However, the epigenomic changes in VSCC remain relatively underexplored.

Epigenetic modifications, or changes in gene expression without alterations in DNA sequences, can play a crucial role in carcinogenesis. Of the several mechanisms of epigenetic modification, DNA methylation, which involves binding of a methyl (-CH3) group to the cytosine residues of the promoter cytosine-phosphate-guanine (CpG) dinucleotides, has been most widely studied. Aberrations of CpG-island methylation have been implicated in the development of several gynecological and non-gynecological cancers [19,20,21,22].

In this exploratory study, we aimed to identify methylation-based biomarkers of potential clinical relevance by investigating the genes that are differentially methylated in VSCC compared to normal vulvar tissue. To this end, we performed genome-wide methylation sequencing on a set of VSCC, and normal vulvar tissues from women with no history of vulvar (pre)malignancies.

## 2. Materials and Methods

### 2.1. Ethical Clearance

This study follows the guidelines of the Dutch Federation of Biomedical Scientific Societies (www.federa.org/codes-conduct, last accessed 18 May 2021), which state that no separate ethical approval is required for the use of anonymized residual tissue procured during regular treatment. Approval for this study was also obtained from the Institutional Review Board of Erasmus MC (MEC-2020-0731).

### 2.2. Tissue Procurement

Cases of primary VSCC treated with curative intent between 2016 and 2018 were identified from the electronic records of the Department of Pathology, Erasmus MC. Normal vulvar tissues were procured from women without any history of vulvar (pre)malignancies, who underwent surgery for benign vulvar pathologies. Hematoxylin-eosin (HE) stained glass-slides and formalin-fixed paraffin embedded (FFPE) tissues were retrieved from the archives.

### 2.3. Tissue Processing

Twelve sections of 4µm thickness were prepared from the FFPE-tissues. The first and the last sections were stained with HE to confirm the presence of the tissue of interest. Eight of the remaining slides were stained with hematoxylin to extract tissue for DNA-isolation, and 2 were used for immunohistochemical staining.

### 2.4. Immunohistochemistry

Immunohistochemistry (IHC) was performed with p16 (CintecR©) and p53 (clone: Bp53-11) on an automated, validated staining system (Ventana Benchmark ULTRA, Ventana Medical Systems, Tucson, AZ, USA) according to manufacturer’s protocol. Expression patterns of p16 and p53 were used to categorize the VSCCs as HPV-associated or HPV-independent. p16 is considered to be a reliable surrogate marker of high-risk HPV-infection, and aberrant patterns of p53-expression have been reported to reflect underlying *TP53* mutations [23,24,25,26].

p16 and p53 were scored following published literature [23,24,25,26], as described below.

p16: block-type expression = continuous, strong, nuclear and/or cytoplasmic expression involving ≥ 1/3rd of epithelial thickness; non-block-type expression = patchy expression in clusters of cells; no expression = complete lack of expression.

p53: mutant patterns = diffuse (basal to parabasal) overexpression/basal overexpression/null-pattern/cytoplasmic expression; wild-type pattern (scattered) = scattered, heterogeneous, basal or parabasal expression; wild-type pattern (mid-epithelial) = heterogeneous, mid-epithelial expression with sparing of basal cells and/or lower parabasal cells.

### 2.5. DNA-Isolation

DNA-isolation was performed on the Maxwell RSC DNA FFPE kit© (Promega Corporation, Madison, WI, USA) following manufacturer’s instructions. In brief, the regions of interest with a minimum lesional cell percentage of 60 were selected on the HE-stained slides. The corresponding areas were microdissected manually from the hematoxylin-stained slides using sterile scalpel blades, and placed in sample tubes. Next, 300 µL of mineral oil was added to the sample tubes, and heated at 80 °C for 2 min, followed by the addition of 250 µL of master mix comprising lysis buffer, proteinase K, and blue dye. The tubes were then centrifuged at 10,000× *g* for 20 s, heated at 56 °C for 30 min and incubated at 80 °C for 4 h. The samples were next incubated with RNaseA for 5 min at room temperature. The concentration of the extracted DNA was measured with the Qubit 2.0 fluorometer (Thermo FisherScientific, Waltham, MA, USA).

### 2.6. Methylation Assay

Bisulfite conversion and measurement of DNA methylation was performed at the Human Genomics Facility (HUGE-F) of Erasmus MC. Bisulfite conversion was performed on 250 ng of genomic DNA extracted from the FFPE samples, using the EZ-96 DNA Methylation deep-well Kit (Zymo Research, Irvine, CA, USA). The samples were plated in a randomized order. Next, restoration was performed on the samples with the Infinium HD FFPE DNA Restore Kit (Illumina Inc., San Diego, CA, USA). Finally, the restored samples were hybridized on the Infinium MethylationEPIC BeadChip (Illumina Inc., San Diego, CA, USA). DNA methylation was measured with the Infinium MethylationEPIC BeadChip (Illumina Inc., San Diego, CA, USA), following manufacturer’s protocol. The Illumina Infinium Methylation EPIC BeadChip Kit (Illumina, San Diego, CA, USA) is a high-throughput array that allows quantification of more than 850,000 methylation sites across the human genome at single nucleotide resolution.

### 2.7. Statistical Analysis

For inclusion in the analysis, the following criteria were used–(i) samples that passed technical quality checks, such as extension, hybridization, and bisulfite conversion according to the criteria set by Illumina, (ii) samples with a call rate of >90%, and (iii) probes with a detection *p*-value > 0.05 or bead number >3 for at least 10% of the samples. Beta (β)-values were used to measure the methylation probe intensity and the overall intensity. Differences in the β-values, or, Δβ-values were used to identify the differentially methylated probes; Δβ can assume any value between 0 and ±1. Methylation data were analyzed using PARTEK© Genomics Suite© software (Version 7.0) Functional normalization of the data was performed. Principal component analysis (PCA) scatter plots were constructed for quality check. Analysis of variance (ANOVA) was performed using M-values to test the statistical significance of the differential methylation; false-discovery rate (FDR) < 0.05 was considered significant. Unsupervised hierarchical clustering was performed to identify the differentially methylated probes. For functional annotation of the differentially methylated genes, (DMGs), DAVID [27,28] and Ingenuity Pathway Analysis (IPA QIAGEN Inc.) were used. In addition, to visualize the ranges of β-values for specific DMGs, box-plots were constructed. Copy number variation (CNV) analysis was performed using the probe intensity data from the iDat files following the methodology described in the PARTEK Genomic Suite Documentation, which can be accessed at this link: https://cutt.ly/WnU0W02 (last accessed 18 May 2021).

## 3. Results

### 3.1. Clinico-Pathological Information

Eighteen VSCC and 6 normal vulvar tissues were included in this study; clinico-pathological characteristics are presented in Table 1. Of the VSCCs, 3 (17%) showed block-type p16-expression, and were categorized as HPV-associated, and 15 (83%) showed non-block-type or no p16-expression, and were categorized as HPV-independent. Of the HPV-independent VSCCs, 13 (87%) showed mutant patterns and 2 (13%) showed wild-type pattern of p53-expression (Table 1).

### 3.2. Differentially Methylated Genes

**Figure 1:**
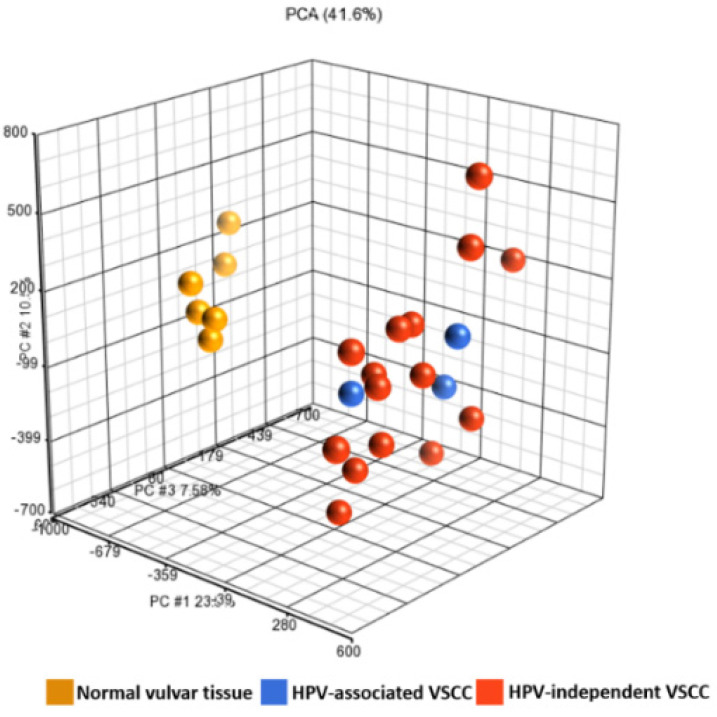
Principal component analysis of the differentially methylated probes shows clear clustering for VSCCs and normal vulvar tissues.

**Figure 2:**
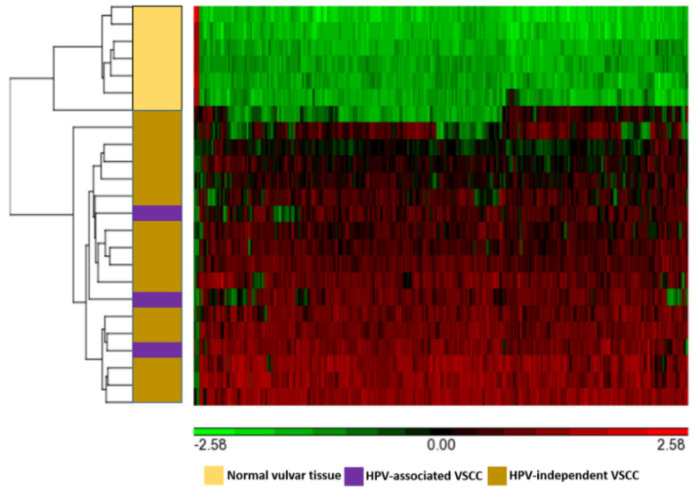
Unsupervised hierarchical clustering of the differentially methylated probes for VSCC and normal vulvar tissues, based on the differences in the β-values (Δβ-values–indicated in the scale). Green represents hypo-methylated, and red represents hyper-methylated probes.

Of the 850,000 methylation probes of Illumina Infinium MethylationEPIC array, 66,069 probes interrogate CpG sites of all known genes as categorized by Illumina. Of these, using the cut-off of false-discovery rate of 0.05 and Δβ of 0.5, 387 CpG-island probes that map to 199 genes were found to be differentially methylated with statistical significance in VSCCs compared to normal vulvar tissues. Of these, 195 genes were hyper-methylated, and 4 genes were hypo-methylated. Using unsupervised hierarchical clustering, clear clustering of the differentially methylated probes was observed for VSCCs and normal vulvar tissues (Figure 1 and Figure 2). However, there was more intra-cluster heterogeneity in VSCCs than in normal vulvar tissues. The top 25 hyper-methylated genes based on the Δβ-values are presented in Table 2, and the complete list of the DMGs is presented in Table S1. The most frequently hyper-methylated genes were ZIC family member 4 (*ZIC4*), coiled-coiled domain containing 181 (*CCDC181*/*C1orf114*), neuroexophilin 1 (*NXPH1*), six homeobox 6 (*SIX6*), zinc finger protein interacting with K protein 1 (*ZIK1*), and zinc finger protein 135 (*ZNF135*).

**Figure 3:**
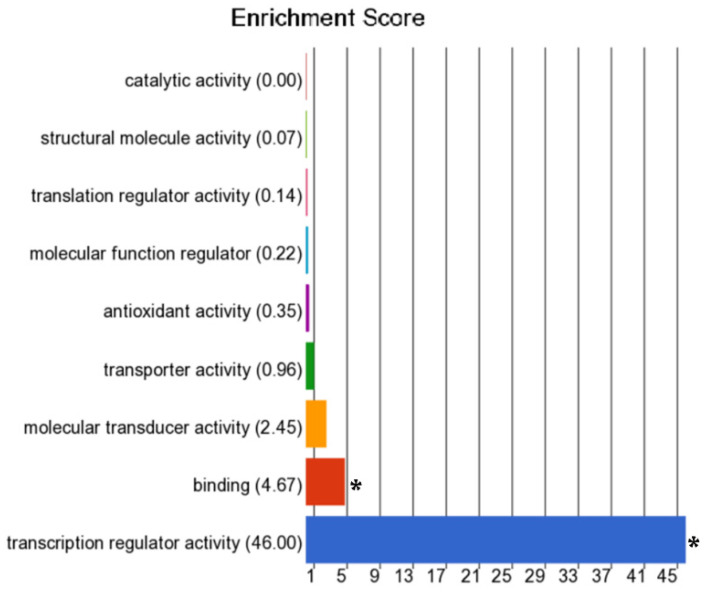
Molecular function of the differentially methylated genes, derived from Gene-Ontology. The x-axis indicates the enrichment scores, and the y-axis indicates the molecular functions. Enrichments that are statistically significant (*p*-value < 0.05) are indicated with asterisks.

As for the molecular function, most of the genes that were hyper-methylated in VSCCs were found to be involved in transcription regulator activity (Figure 3). The majority of the DMGs were also significantly associated with non-melanoma solid tumors, non-hematologic malignant neoplasms, extracranial solid tumors, and carcinoma. Based on the z-scores, activity of biological process involved in the regulation of organismal death was found to be significantly increased and those involved in the regulation of the quantity of cells were found be significantly decreased. Complete list of molecular functions, disease processes, and biological processes enriched by the DMGs, along with the *p*-values and enrichment scores are presented in Tables S2–S4.

### 3.3. Copy Number Variations

**Figure 4:**
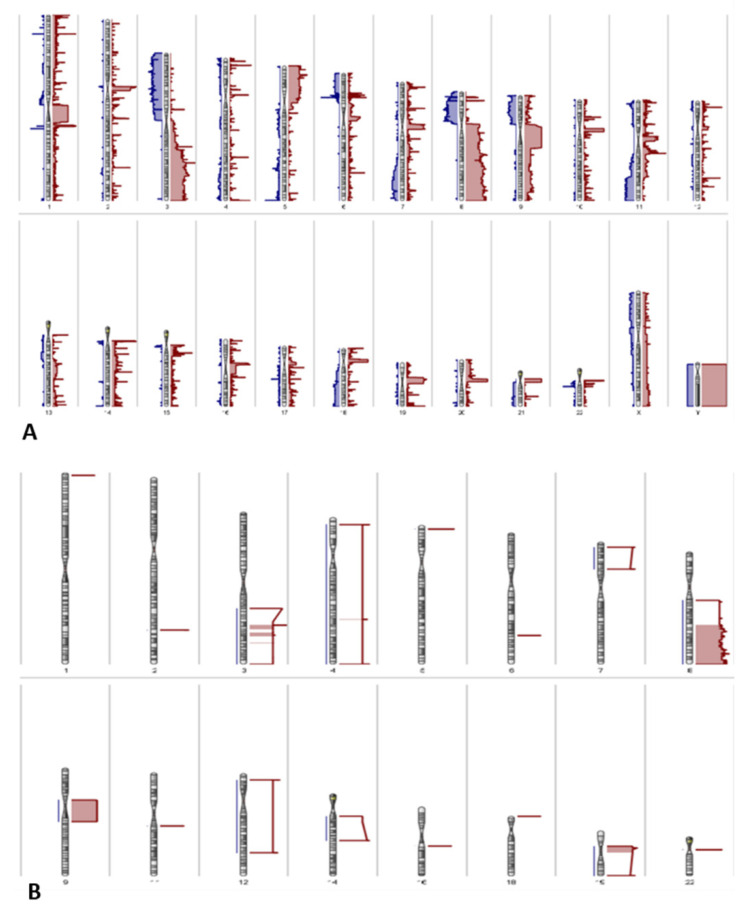
Karyogram depicting the amplifications (red) and deletions (blue) detected in (**A**) all VSCC samples, frequent amplifications in VSCC, (**B**) in >50% of VSCC samples.

Both amplifications and deletions of chromosomal regions were present in VSCCs. Amplifications in chromosomes 3, 8, and 9 were most frequently detected; these were present in >50% of the VSCC samples (Figure 4).

### 3.4. Integration of Differential Methylation and Copy Number Variation Analyses

**Figure 5:**
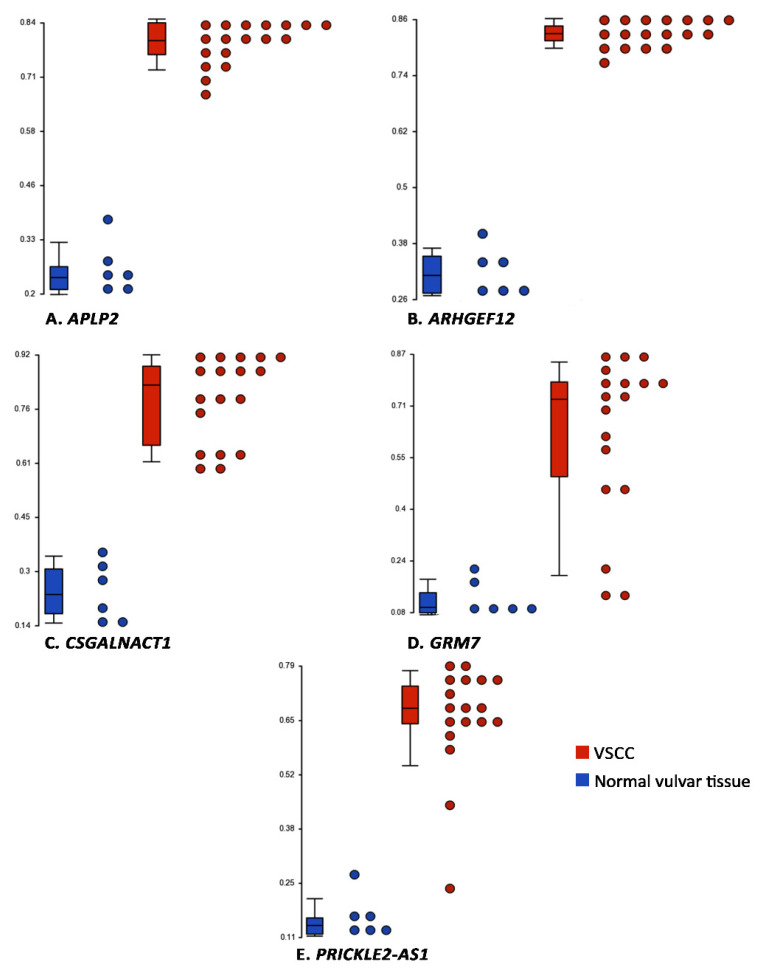
Box-plots depicting the range of β-values in normal vulvar tissues and VSCC, for the 5 genes that had a deletion in one allele and hyper-methylation in another allele. The y-axes indicate the β-values, horizontal lines in the boxes represent the medians, and the whiskers represent the 5th and 95th percentiles.

Next, we sought to identify genes that had hyper-methylation in one allele and deletion in another allele. For this, we performed an overlay analysis of all the hyper-methylated probes (*n* = 631; this included both CpG-island and non-CpG-island probes), and genes that were deleted in at least 6 samples of VSCC and not amplified in any sample of VSCC. A set of 5 such genes were identified, namely amyloid beta precursor-like protein 2 (*APLP2*), Rho guanine nucleotide exchange factor 12 (*ARHGEF12*), chondroitin sulfate N-acetylgalactosaminyltransferase 1 (*CSGALNACT1*), glutamate metabotropic receptor 7 (*GRM7*), and PRICKLE2 antisense RNA 1 (*PRICKLE2-AS1*). The ranges of the β-values of these genes in VSCC and in normal vulvar tissues is presented in Figure 5, and their molecular functions and the associated canonical pathways are presented in Table S5.

## 4. Discussion

In recent years, characterization of the genome and epigenome has provided novel insight into the carcinogenesis of several cancers, and has facilitated diagnosis and tailoring of therapeutic strategies. Genomic changes in the form of somatic mutations can alter gene expression and drive the carcinogenesis. For VSCC and its precursor lesion, vulvar intraepithelial neoplasia (VIN), somatic mutations have been investigated in several studies using targeted (amplicon panel-based) sequencing, or whole exome sequencing [7,10,11,12,18,29,30,31,32,33]. However, for comprehensive understanding of the carcinogenesis, characterization of epigenetic changes is also crucial. Unfortunately, for VSCC these changes have been interrogated in only a few studies [34,35,36,37,38,39,40].

In this study, we used a high-throughput genome-wide methylation array to identify genes that are differentially methylated in VSCC compared to normal vulvar tissue. DNA methylation is the most well studied epigenetic mechanism, and is known to play a critical role in repressing gene expression and maintaining genomic stability. We identified a set of 199 DMGs for VSCC; of these, the majority (*n* = 194) were hyper-methylated; hyper-methylation is considered to down-regulate gene expression.

Interestingly, on unsupervised hierarchical clustering, distinct clusters of differentially methylated probes were identified for VSCC and for normal vulvar tissue. However, these probes were more heterogeneous for VSCCs than for normal vulvar tissues. Intra-and inter-tumoral heterogeneity in terms of mutational and copy number variation profile of VSCCs have been previously reported [26,33]. Our results indicate the existence of inter-tumoral heterogeneity in the methylation profiles of VSCC. We did not observe any specific clustering corresponding to the HPV-status of VSCC; however, this could be due to the smaller number of HPV-associated VSCCs in this study.

Of the DMGs that were detected for VSCCs, *ZIC1*, *PRDM14*, *SST*, *LHX8*, and *ZNF582* have been previously detected in HPV-associated SCC of the cervix and the anus, and in their respective precursor lesions [41,42]. We observed that *ZIC1* was hyper-methylated in both subtypes of VSCC, indicating an involvement of this gene in the HPV-independent pathway of VSCC carcinogenesis as well. A recent study reported an association between increasing methylation levels of *ZIC1* and the risk of progression of VIN to VSCC [40]. One of the hyper-methylated DMGs in our study, *DNMT3A*, has been previously detected in VSCC, and was reported to correlate with a risk of recurrence [37]. *DNMT3A* is a de-novo methyltransferase, which is considered to bring about silencing of some critical genes during tumorigenesis, including tumor suppressor genes, and genes involved in cell cycle regulation, transcription regulation, and signal transduction [43]. A previous study detected hyper-methylation of *IRF6* in VSCC; this was however, not detected in our study [44].

Most of the genes that we found to be hyper-methylated in VSCCs are involved in transcription regulator activity, indicating that disruption of this process may play a vital role in VSCC carcinogenesis. Since hyper-methylation of the promoters of regulatory genes is known to result in activation of oncogenes and inactivation of tumor suppressor genes, proportion of hyper-methylated genes tends to be higher in cancerous tissue compared to healthy tissues from the same anatomical site. This has been observed in both HPV-associated cancers, e.g., cervical SCC, and in HPV-independent cancers, e.g., oral and laryngeal SCC [45,46]. Studies on cervical SCC indicate that the HPV-oncoproteins E6 and E7 activate DNA methyltransferases, which promote the hyper-methylation of CpG-islands and silencing of host genes, including *CDH1*, *TP53*, *RASSF1,* and *CDKN2A* [45]. It could be investigated whether the HPV-oncoproteins cause gene silencing in HPV-associated VSCCs using a similar mechanism.

As the Infinium EPIC BeadChip array is essentially a dense single nucleotide polymorphism array, the data generated from the array sequencing can also be used to identify copy number variations, therefore allowing integration of the genetic and epigenetic data [47]. We also observed amplifications of chromosomes 3, 8, and 9, to be present in >50% of the VSCCs; amplification of chromosomes 3 and 8 has also been previously detected in cervical SCC and VSCC [5,41]. Finally, we identified a set of 5 genes that were hyper-methylated, as well as, frequently deleted in VSCC. Of these somatic mutations of *APLP2* and *ARGHEF12* have been detected in head-and-neck SCC and cervical SCC respectively, and that of *GRM7* and *GALNACT* have been detected in endometrial carcinoma [48,49,50].

One of the strengths of this exploratory study is the use of a high-throughput genome-wide methylation sequencing array to interrogate the methylation profiles of VSCC and normal vulvar tissue. The Methylation EPIC BeadChip array is one of the most comprehensive methods currently available for methylation profiling of human tissues. Using this array allowed us to identify some novel DMGs for VSCC in our exploratory study. The control tissues used in this study were derived from age-matched women with no history of vulvar (pre)malignancies. This offers a unique advantage in that these tissues are unlikely to harbor background molecular changes that may be present in paired normal tissues obtained from patients with cancers, which are often used as controls in molecular studies. However, the lack of such paired normal tissues in this study may also be a potential limitation, as molecular analysis of these tissues could provide insight into the underlying changes in the carcinogenic field. Furthermore, this study was limited by the small sample size, as a result of which heterogeneity in the methylation profiles of VSCCs, or the correlation of the methylation and clinical data could not be interrogated by performing sub-group analysis. Validation of our results in independent larger cohorts and correlating the methylation data with gene expression analyses will be important. Valuable information can be obtained from performing immunohistochemistry and/or quantitative methylation-specific polymerase chain reaction for the specific DMGs. Recent studies suggest that methylation changes are an early event in VSCC carcinogenesis. Therefore, methylation levels of the DMGs identified in this study in the precursor lesions of VSCC or VIN, could be investigated. Future studies could interrogate whether these methylation changes in VSCCs are also detectable in the circulating tumor DNA, to identify potential biomarkers for liquid biopsy.

## 5. Conclusions

We identified a set of DMGs in VSCC in this study with a view to promote hypothesis-generation, and to provide a resource for future investigations on the diagnostic, prognostic, predictive, or therapeutic potential of methylation-based biomarkers.

## Figures and Tables

**Table 1:** Clinico-pathological characteristics.

Characteristics	Diagnoses		
	HPV-Independent VSCC (*n* = 15)	HPV-Associated VSCC (*n* = 3)	Normal Vulvar Tissue (*n* = 6)
Median Age (Range) in years	76 (52–90)	73 (65–81)	75 (52–90)
Location			
Vulvar skin with adnexa	8 (53)	1 (33)	1 (17)
Vulvar skin without adnexa	7 (47)	2 (67)	5 (83)
Treatment			
Excision with lymph node dissection/sentinel node procedure	14 (94)	3 (100)	0 (0)
Excision only	1 (6)	0 (0)	6 (100)
Follow-up			
No evidence of VIN/VSCC	7 (47)	2 (67)	6 (100)
VIN	1 (6)	0 (0)	0 (0)
VSCC recurrence	6 (41)	1 (33)	0 (0)
Died	1 (6)	0 (0)	0 (0)
p16-expression			
Block-type	0 (0)	3 (100)	0 (0)
Non-block-type	8 (53)	0 (0)	1 (17)
No expression	7 (47)	0 (0)	5 (83)
p53-expression			
Diffuse overexpression	9 (60)	1 (33)	0 (0)
Basal overexpression	1 (7)	0 (0)	0 (0)
Null pattern	3 (20)	0 (0)	0 (0)
Wild-type expression (scattered)	2 (13)	0 (0)	6 (100)
Wild-type expression (mid-epithelial)	0 (0)	2 (67)	0 (0)

**Table 2:** Differentially methylated genes based on Δβ-values (top 25).

Symbol	Entrez Gene Name	Δβ-Values	Location	Type(s)
*C1QTNF4*	C1q and TNF related 4	0.698	Extracellular Space	other
*TNR*	tenascin R	0.676	Plasma Membrane	other
*ZSCAN1*	zinc finger and SCAN domain containing 1	0.67	Nucleus	transcription regulator
*ZNF135*	zinc finger protein 135	0.662	Nucleus	transcription regulator
*ZNF471*	zinc finger protein 471	0.66	Nucleus	transcription regulator
*CCDC8*	coiled-coil domain containing 8	0.63	Plasma Membrane	other
*TBX3*	T-box transcription factor 3	0.626	Nucleus	transcription regulator
*LOC728392*	uncharacterized LOC728392	0.625	Other	other
*SSTR1*	somatostatin receptor 1	0.621	Plasma Membrane	G-protein coupled receptor
*MARCHF11*	membrane associated ring-CH-type finger 11	0.617	Other	other
*UNC79*	unc-79 homolog, NALCN channel complex subunit	0.615	Other	other
*ACSF2*	acyl-CoA synthetase family member 2	0.614	Cytoplasm	enzyme
*ZIK1*	zinc finger protein interacting with K protein 1	0.613	Other	other
*KCNC2*	potassium voltage-gated channel subfamily C member 2	0.609	Plasma Membrane	ion channel
*PABPC5*	poly(A) binding protein cytoplasmic 5	0.607	Cytoplasm	other
*PPFIA3*	PTPRF interacting protein alpha 3	0.604	Plasma Membrane	phosphatase
*BARHL2*	BarH-like homeobox 2	0.603	Nucleus	transcription regulator
*BOLL*	boule homolog, RNA binding protein	0.603	Cytoplasm	translation regulator
*ONECUT2*	one cut homeobox 2	0.601	Nucleus	transcription regulator
*HAS1*	hyaluronan synthase 1	0.6	Plasma Membrane	enzyme
*CMTM2*	CKLF-like MARVEL transmembrane domain containing 2	0.596	Extracellular Space	cytokine
*ZIC4*	Zic family member 4	0.596	Nucleus	transcription regulator
*LBX2*	ladybird homeobox 2	0.595	Nucleus	transcription regulator
*SALL1*	spalt-like transcription factor 1	0.592	Nucleus	transcription regulator

## Data Availability

The datasets generated by performing whole-genome methylation sequencing are available for sharing with physicians and researchers for research purposes. Data will be shared upon reasonable request following our hospital guidelines. Requests should be directed to the corresponding author.

## Supplementary Materials

The following are available online at https://www.mdpi.com/article/10.3390/cancers13143580/s1, Table S1: Complete list of all differentially methylated genes; Table S2: List of enriched molecular functions; Table S3: List of enriched disease processes; Table S4: List of enriched biological processes; Table S5: Functional annotations of the genes that are hyper-methylated and deleted in vulvar squamous cell carcinoma.
