# Supplementary material for: Exploring Differentially Methylated Genes in Vulvar Squamous Cell Carcinoma
Source: Cancers (Basel). 2021 Jul 16;13(14):3580. doi: 10.3390/cancers13143580 (PMC8306700; doi:10.3390/cancers13143580)
Supplement: Supplementary file 1 [file cancers-13-03580-s001.zip › cancers-1245370-supplementary/Table_S5.pdf]

Supplement 2

Table S2: Functional annotations of the genes that are hyper-methylated and deleted in vulvar squamous cell carcinoma

Entrez name	Summary	Molecular function	Biological process	Cellular component	Mutations
amyloid beta precursor like protein 2	This gene encodes amyloid precursor- like protein 2 (APLP2), which is a member of the APP (amyloid precursor protein) family including APP, APLP1 and APLP2. This protein is ubiquitously expressed. It contains heparin-, copper- and zinc- binding domains at the N-terminus, BPTI/Kunitz inhibitor and E2 domains in the middle region, and transmembrane and intracellular domains at the C-terminus. This protein interacts with major histocompatibility complex (MHC) class I molecules. The synergy of this protein and the APP is required to mediate neuromuscular transmission, spatial learning and synaptic plasticity. This protein has been implicated in the pathogenesis of Alzheimer's disease. Multiple alternatively spliced transcript variants encoding different isoforms have been identified. [provided by RefSeq, Aug 2011]	NA binding; heparin binding; identical protein binding; metal ion binding; protein binding; serine-type endopeptidase inhibitor activity; transition metal ion binding	cellular copper ion homeostasis; cellular protein metabolic process; cholesterol metabolic process; embryo development; extracellular matrix organization; forebrain development; G-protein coupled receptor signaling pathway; locomotory behavior; mating behavior; midbrain development; negative regulation of endopeptidase activity; neuromuscular process controlling balance; platelet degranulation; post-translational protein modification; regulation of	endoplasmic reticulum lumen; extracellular vesicular exosome; integral to membrane; membrane; nucleus; plasma membrane; platelet alpha granule membrane	Somatic missense mutations in head and neck SCC

			epidermal growth factor-activated receptor activity; regulation of protein binding; suckling behavior		
Rho guanine nucleotide exchange factor 12	Canonical pathways – Actin Cytoskeleton Signaling; Axonal Guidance Signaling; Breast Cancer Regulation by Stathmin1; Molecular Mechanisms of Cancer; Phospholipase C Signaling; Reelin Signaling in Neurons; RhoA Signaling; RhoGDI Signaling; Semaphorin Neuronal Repulsive Signaling Pathway; Semaphorin Signaling in Neurons; Signaling by Rho Family GTPases; Thrombin Signaling<br><br>Rho GTPases play a fundamental role in numerous cellular processes that are initiated by extracellular stimuli working through G protein-coupled receptors. The encoded protein may form a complex with G proteins and stimulate Rho-dependent signals. This protein has been observed to form a myeloid/lymphoid fusion partner in acute myeloid leukemia. Three transcript variants encoding	G-protein coupled receptor binding; GTPase activator activity; guanyl-nucleotide exchange factor activity; protein binding; Rho guanyl-nucleotide exchange factor activity	G-protein coupled receptor signaling pathway; intracellular signal transduction; positive regulation of apoptotic process; positive regulation of GTPase activity; regulation of Rho protein signal transduction; regulation of small GTPase mediated signal transduction; Rho protein signal transduction	cellular membrane, Cytoplasm, cytosol, perikaryon, plasma, Plasma Membrane	Somatic mutations in cervical SCC

	different isoforms have been found for this gene. [provided by RefSeq, Jul 2014]				
chondroitin sulfate N-acetylgalactosaminyltransferase 1	Canonical pathways – Chondroitin and Dermatan Biosynthesis; Chondroitin Sulfate Biosynthesis; Chondroitin Sulfate Biosynthesis (Late Stages); Dermatan Sulfate Biosynthesis; Melatonin Degradation I; Nicotine Degradation II; Nicotine Degradation III; Serotonin Degradation; Superpathway of Melatonin Degradation; Thyroid Hormone Metabolism II (via Conjugation and/or Degradation)	acetylgalactosaminyl transferase activity; glucuronosyl-N-acetylgalactosaminyl-proteoglycan 4-beta-N-acetylgalactosaminyl transferase activity; glucuronosyltransferase activity; glucuronylgalactosyl proteoglycan 4-beta-N-acetylgalactosaminyl transferase activity; metal ion binding; peptidoglycan glycosyltransferase activity; transferase activity	cartilage development; chondroitin sulfate biosynthetic process; chondroitin sulfate metabolic process; chondroitin sulfate proteoglycan biosynthetic process; chondroitin sulfate proteoglycan biosynthetic process, polysaccharide chain biosynthetic process; dermatan sulfate proteoglycan biosynthetic process; endochondral ossification; extracellular matrix organization; heparan sulfate proteoglycan biosynthetic process, polysaccharide chain biosynthetic process; heparin	Golgi apparatus; Golgi cisterna membrane; Golgi membrane; integral to Golgi membrane; integral to membrane; membrane	Somatic mutaions in endometrioid carcinoma

			biosynthetic process; proteoglycan biosynthetic process; UDP-glucuronate metabolic process; UDP-N-acetylgalactosamine metabolic process		
glutamate metabotropic receptor 7	L-glutamate is the major excitatory neurotransmitter in the central nervous system, and it activates both ionotropic and metabotropic glutamate receptors. Glutamatergic neurotransmission is involved in most aspects of normal brain function and can be perturbed in many neuropathologic conditions. The metabotropic glutamate receptors are a family of G protein-coupled receptors that have been divided into three groups on the basis of sequence homology, putative signal transduction mechanisms, and pharmacologic properties. Group I includes GRM1 and GRM5, and these receptors have been shown to activate phospholipase C. Group II includes GRM2 and GRM3, while Group III includes	adenylate cyclase inhibiting G-protein coupled glutamate receptor activity; adenylate cyclase inhibitor activity; calcium channel regulator activity; calcium-dependent protein binding; calcium ion binding; calmodulin binding; glutamate binding; glutamate receptor activity; G-protein coupled receptor activity; group III metabotropic glutamate receptor activity; identical protein binding; PDZ domain binding; protein binding;	adenylate cyclase-inhibiting G-protein coupled glutamate receptor signaling pathway; adenylate cyclase-inhibiting G-protein coupled receptor signaling pathway; adult behavior; associative learning; behavioral fear response; calcium ion transmembrane transport; conditioned taste aversion; G-protein coupled glutamate receptor signaling pathway; G-protein coupled receptor signaling pathway; learning or memory;	asymmetric synapse; axon; axon terminus; cell cortex; cell surface; dendrite; dendritic shaft; Golgi apparatus; integral to membrane; integral to plasma membrane; macromolecular complex; membrane; neuronal cell body; plasma membrane; postsynaptic membrane; presynaptic active zone; presynaptic active zone membrane; presynaptic membrane; receptor complex; rough endoplasmic	Somatric missense mutations in endometrial carcinoma

	GRM4, GRM6, GRM7 and GRM8. Group II and III receptors are linked to the inhibition of the cyclic AMP cascade but differ in their agonist selectivities. Multiple transcript variants encoding different isoforms have been found for this gene. [provided by RefSeq, Jun 2009]	serine binding; voltage-gated calcium channel activity	memory; multicellular organismal response to stress; negative regulation of glutamate secretion; negative regulation of neuron apoptotic process; neurological system process; regulation of cyclase activity; regulation of synaptic transmission, glutamatergic; regulation of synaptic vesicle exocytosis; response to stimulus; sensory perception of sound; short-term memory; signal transduction; synaptic transmission; transmission of nerve impulse	reticulum; synapse; terminal button
PRICKLE2 antisense RNA 1	Not characterized			
